# BILATERAL TEMPOROMANDIBULAR JOINT DISLOCATION AND ANTIPSYCHOTIC TREATMENT: A CASE REPORT

**DOI:** 10.1192/j.eurpsy.2023.2156

**Published:** 2023-07-19

**Authors:** R. Obrador i Font, S. Villa Hoz, M. Urretavizcaya Sarachaga

**Affiliations:** 1 Psychiatry, Hospital Universitari Bellvitge, L’Hospitalet de Llobregat, Spain

## Abstract

**Introduction:**

Acute dystonia is a type of extrapyramidal effect that is produced by the blockade of dopaminergic D2 receptors typical of antipsychotics. There is a subtype acute dystonia called oromandibular, which produces perioral manifestations. In extreme cases it can even produce temporomandibular joint dislocation, bilateral being more frequent than unilateral. In this abstract it is presented the clinical case of a 22-year-old female who attended to the Emergency Department due to a bilateral temporomandibular joint dislocation that was finally attributed to antipsychotic treatment.

**Objectives:**

The objective of the clinical case is to point out the importance of examination and clinical history for psychiatric diagnosis.

**Methods:**

Review of various scientific articles related to acute dystonia.

**Results:**

It is a report of a 22-year-old female with no medical-surgical or psychiatric history who was imprisoned for legal conflicts. During her stay in prison, she presented reactive depressive and anxiety symptoms, receiving antidepressant and anxiolytic treatment. After two months in prison, she was released and, two days after her release, she attended to the Emergency Department due to rigid akinetic symptoms, drowsiness, mutism and urination difficulties. Complementary tests revealed bilateral temporomandibular joint dislocation, with no other organicity wich could justify the rest of the symptoms, so she was admitted to the Acute Psychiatry Unit for study.

During her admission, the physical examination (akinetic rigid picture, muscle contraction and galactorrhea) raised the possibility that it was extrapyramidal symptomatology secondary to antipsychotic treatment. Given that suspicion, intramuscular biperiden 5 mg/ml was administered, improving the condition in two hours. In a second time, the initial anamnesis was redone; the patient added that during her stay in prison she had presented psychomotor agitation for which she had recieved an intramuscular treatment that she was not able to specify. All this information confirmed the initial suspicion; it was extrapyramidal symptomatology induced by antipsychotic treatment. Thus, treatment with oral biperiden 4 mg/12 hours was continued and the condition completely remitted in five days.

**Conclusions:**

In this abstract it is presented the case of a bilateral temporomandibular joint dislocation induced by antipsychotic treatment. Although it is a rare presentation, other cases like that have been described in the literature, specifically with the use of haloperidol, risperidone, amisulpride and aripiprazole. Given the high frequency of adverse effects of antipsychotics, it is essential that psychiatrists remain trained in their prediction and management.

**Disclosure of Interest:**

None Declared

